# Intravenous methylprednisolone or immunoglobulin for anti-glutamic acid decarboxylase 65 antibody autoimmune encephalitis: which is better?

**DOI:** 10.1186/s12868-020-00561-9

**Published:** 2020-03-30

**Authors:** Tao-Ran Li, Yu-Di Zhang, Qun Wang, Xiao-Qiu Shao, Zhi-Mei Li, Rui-Juan Lv

**Affiliations:** 1 Department of Neurology, Beijing Tiantan Hospital, Capital Medical University; China National Clinical Research Center for Neurological Diseases, 119 South Fourth Ring West Road, Fengtai District, Beijing, 100070 People’s Republic of China; 2 Department of Neurology, Xuanwu Hospital of Capital Medical University, 45 Chang Chun Road, Xicheng District, Beijing, 100053 People’s Republic of China; 3 Department of Neurology, the Second Hospital of Hebei Medical University, Hebei Medical University, 215 Heping West Road, Xinhua District, Shijiazhuang, 050000 People’s Republic of China

**Keywords:** Glutamic acid decarboxylase 65, Autoimmune encephalitis, Methylprednisolone, Immunoglobulin

## Abstract

**Background:**

Patients positive for anti-glutamic acid decarboxylase 65 (GAD65) antibodies have attracted increasing attention. Their clinical manifestations are highly heterogeneous and can be comorbid with tumors. Currently, there is no consensus on the therapeutic regimen for anti-GAD65-associated neurological diseases due to the clinical complexity, rarity and sporadic distribution. We reported six anti-GAD65 autoimmune encephalitis (AE) patients who received intravenous methylprednisolone (IVMP) or immunoglobulin (IVIG) or both. Then, we evaluated the therapeutic effect of both by summarizing results in previous anti-GAD65 AE patients from 70 published references.

**Results:**

Our six patients all achieved clinical improvements in the short term. Unfortunately, there was no significant difference between IVMP and IVIG in terms of therapeutic response according to the previous references, and the effectiveness of IVMP and IVIG was 45.56% and 36.71%, respectively. We further divided the patients into different subgroups according to their prominent clinical manifestations. The response rates of IVMP and IVIG were 42.65% and 32.69%, respectively, in epilepsy patients; 60.00% and 77.78%, respectively, in patients with stiff-person syndrome; and 28.57% and 55.56%, respectively, in cerebellar ataxia patients. Among 29 anti-GAD65 AE patients with tumors, the response rates of IVMP and IVIG were 29.41% and 42.11%, respectively. There was no significant difference in effectiveness between the two regimens among the different subgroups.

**Conclusion:**

Except for stiff-person syndrome, we found that this kind of AE generally has a poor response to IVMP or IVIG. Larger prospective studies enrolling large numbers of patients are required to identify the optimal therapeutic strategy in the future.

## Background

Glutamic acid decarboxylase 65 (GAD65), an intracellular antigen that is highly expressed in pancreatic β-cells and the presynaptic terminal of inhibitory neurons, is becoming increasingly important both clinically and experimentally [1–4]. Patients with high serum and cerebrospinal fluid (CSF) titers of anti-GAD65 antibodies (Abs) have been proven to be clinically heterogeneous, including those with stiff-person syndrome (SPS), cerebellar ataxia (CA), limbic encephalitis (LE), nonlimbic autoimmune encephalitis (AE), autonomic neuropathy and other multifarious neurological disorders [5, 6]. Furthermore, tumors have been identified in several cases, indicating that anti-GAD65 Abs are associated with paraneoplastic neurological syndrome [7]. Even more perplexing, anti-GAD65 Abs are frequently comorbid with one or more systemic autoimmune diseases and other Abs [5, 8, 9]. Because of the clinical heterogeneity, together with highly variable complexity, low prevalence and sporadic distribution, performing large-scale clinical trials are challenging. Therefore, there is currently no global consensus on the therapeutic regimen, mainly including corticosteroids, immunoglobulin, plasma exchange and other immunosuppressant drugs; all of the available experience is based on case reports. However, management may vary widely among different medical centers, even when opposing recommendations are made [9–11].

In this study, we retrospectively analyzed the clinical characteristics of six anti-GAD65 AE patients in our tertiary epilepsy center who presented with different clinical injury sites and severity. To date, no study has indicated which treatment is better for anti-GAD65 AE patients when comparing intravenous methylprednisolone (IVMP) and intravenous immunoglobulin (IVIG). Thus, we analyzed the chosen treatments for all anti-GAD65 AE patients by searching PubMed, and we compared the efficacy of IVMP with that of IVIG. The purpose of the study is to raise the awareness of this disease and guide clinical therapies.

## Patients and Methods

Six subjects from Beijing Tiantan Hospital gave written informed consent for participation and written consent to permit the publication of clinical details. The study was approved by the Medical Ethics Committee of Beijing Tiantan Hospital, Capital Medical University, and was carried out in accordance with the Declaration of Helsinki.

We conducted a search on PubMed for articles up to April 2019 and used the title/abstract search terms “encephalitis” and “GAD” or “glutamic acid decarboxylase”. A total of 133 references were retrieved. The criteria for enrollment were as follows: patients with anti-GAD65 Abs received a therapy of IVMP or IVIG or a combination of both, and we were able to obtain the response to the treatment. We were not particularly concerned about whether patients used antiepileptic drugs (AEDs) because the effect of AEDs was very limited [12, 13]. IVMP or IVIG meant that the patients received high-dose methylprednisolone or immunoglobulin pulse therapy, respectively, but the duration was variable, often by more than 5 days. Reference screening was conducted by two experienced neurological doctors, Tao-Ran Li and Rui-Juan Lv. Forty-nine articles, including 38 case (series) reports and 11 research articles, were included on the basis of their relevance to the inclusion criteria. In addition, a back-search of reference lists from retrieved publications was also conducted to identify other potentially relevant articles. As a result, 21 other articles, including 13 case (series) reports and 8 research articles, were also included.

We eliminated many references for the following reasons. First, we excluded studies for which it was difficult to assess the efficacy of methylprednisolone, immunoglobulin or a combination of methylprednisolone and immunoglobulin because patients received immunosuppressive therapy in addition to IVMP or IVIG meanwhile, such as plasma exchange [14–16], immunoadsorption [17], azathioprine [18], rituximab [19], mycophenolate mofetil [20] or others [21]. Second, we excluded studies for which we could not obtain the patients’ response to IVMP or IVIG or their combination because of the ambiguous descriptions or the inaccessible follow-up information in the original literature [22–27]. Third, we excluded studies for which the different clinical manifestations of patients responded inconsistently to treatment; for example, in patient D in the literature [28], seizure frequency did not respond to IVMP, while CA improved. Fourth, we excluded studies for which patients took prednisone or dexamethasone only orally [19, 29–33]. Fifth, we excluded those patients with concurrent Rasmussen encephalitis [34, 35].

The 70 references included in the statistics are listed in Additional file 1. We marked the references containing patients who presented with seizures, SPS or CA and references containing patients who had coexisting tumors or received combination therapy.

## Statistical analysis

We counted the number of patients who responded to IVMP or IVIG or a combination of both in the literature. At the same time, we also counted the number of patients who did not respond to IVMP, IVIG or a combination of both. The clinical treatment for anti-GAD65 AE patients was diverse and complex, and most patients underwent a variety of treatment options. In many cases, patients changed into the second treatment plan since the first was ineffective or because of illness relapse; therefore, the same patient was likely to be assigned two statistical data points. For example, if one patient made no response to IVMP while responding to IVIG, we counted once that IVIG was effective and counted once that IVMP was ineffective. If one patient responded to combination therapy (not including that there was a time interval between IVMP and IVIG), we counted that combination therapy was effective once instead of calculating once separately that both IVMP and IVIG were effective. If one patient was unresponsive to combination therapy, we counted once separately that both IVMP and IVIG were ineffective. We completely followed the information obtained in the literature and objectively collected data according to the original authors’ standpoints.

Statistical analyses were performed with SPSS 22.0 software (SPSS, Chicago, IL, USA). Differences were evaluated by the Chi squared test or Fisher’s exact test. *P *< 0.05 was considered to be statistically significant.

## Results

### Patients from our center

Six illustrative anti-GAD65 AE cases from our center are comparatively described in Table 1, and we include two typical examples for a detailed description below owing to spatial confinement. The six patients were all right-handed and were born at term to nonconsanguineous Chinese parents, with no abnormal antenatal or postnatal issues of note, and reached developmental milestones appropriately. Except for patients 2, 5 and 6, their past medical histories were unremarkable, and their family histories of seizures or neurological and immune disorders were also not special except that of patient 2. They all presented seizures as the initial symptom; however, their intracranial lesions and subsequent clinical manifestations were different as follows: patient 1 was characterized as having typical LE, patient 2 had obvious brain atrophy and features of multiple different clinical syndromes due to a long-term disease course, the remaining four patients mainly presented with medial temporal lobe injury in imaging (except patient 3), and all patients were characterized as having drug-resistant epilepsy (patient 4 also had SPS). The average time from onset to immunotherapy for the six patients was approximately 3 months, 18 years, 20 months, 19 years, 10 years and 5 years, respectively. They received different treatment regimens, all showing various degrees of improvement in the short term.

**Table 1:** Clinical characteristics of six GAD65 positive-AE patients from our center

	Patient 1	Patient 2	Patient 3	Patient 4	Patient 5	Patient 6
Age/gender	35/F	35/M	29/F	49/F	27/F	33/F
Past history	Unremarkable	Excision of epileptogenic focus in 2015	Unremarkable	Unremarkable	Excision of thymoma in 2013	L Thyroidectomy
Family history	Unremarkable	Hyperthyroidism of his mother	Unremarkable	Unremarkable	Unremarkable	Unremarkable
Symptom onset	2018/08	2000/?	2016/06	2000/?	2009/?	2014/?
Initial symptoms	Seizure	Seizure	Seizure	Seizure	Seizure	Seizure
Seizure types	SPS; CPS; sGTCS	sGTCS; CPS	SPS	CPS; sGTCS	CPS; sGTCS	CPS
Cognition/MoCA	Impaired/22	Impaired/22	Impaired/NA	Impaired/19	Impaired/23	Impaired/23
Other Symptoms	CI	CI, SPS^*^, CA, NBC	CI	CI, SPS^*^	CI	CI
AEDs	LEV, 1000 mg/day	LEV, 1000 mg/day; CBZ, 600 mg/day; CZP, 1 mg/day	CBZ, 500 mg/day	CZP, 4 mg/q8h; CBZ, 400 mg/qd	OXC, 300 mg/q12h; LEV, 250 mg/q12h	LEV, 625 mg/q12h; discontinuation by herself later
Seizure Frequency at the time of Immunotherapy	CPS, 2–3/day; sGTCS, 1–2/month	sGTCS, zero; CPS, 1/day	SPS, 2–6/week	CPS, 2–4/week; sGTCS, 1–2/month	CPS, 1–2/day; sGTCS, 1–2/year	CPS, 2–13/day
MRI lesions	R MTL, Amy, Hipp	Atrophy of L MTL, Hipp and B cerebellum	B FL	B MTL	B Hipp	L Hipp and Amy
^18^F-FDG-PET/CT	Hypometabolism of R TL	NA	Hypometabolism of R Tha, L Hipp and TL	Hypometabolism of L TL	Hypometabolism of L TL	Hypometabolism of L TL
Others	Thymus residue	No	Thymus residue	No	Thymoma	No
Immunotherapy	2018/11	2018/11	2018/02	2019/03	2019/07	2019/04
Comorbidities	No	Hyperthyroidism, LADA	No	No	MG; hyperthyroidism	No
Serum examination showing positive Abs	No	ANA, anti-mitochondrial M2 subtype abs, anti-Ro-52 abs, TPA, TGA, anti-cardiolipin abs	TPA, TGA	No	TPA	No
Anti-GAD65 Abs, serum	1:10 positive	1:100 positive	NA, positive	1:320 positive	1:100 positive	1:100 positive
Anti-GAD65 Abs, CSF	1:100 positive	1:100 positive	NA, positive	1:320 positive	1:320 positive	1:100 positive
Immunotherapy	IVMP and prednisone tapered; IVIG	IVIG twice, and MF	IVIG and prednisone tapered	IVMP and IVIG, medrol tapered	IVMP and IVIG, prednisone tapered	IVMP and IVIG, prednisone tapered
Outcome (2019.07)	CPS, 2–3/month	CPS, 3–4/month	NA	no more seizures; SPS^*^ improved	no more CPS and sGTCS	CPS, 1–2/day

*GAD65* glutamic acid decarboxylase 65, *AE* autoimmune encephalitis, *F* female, *M* male, *SPS* simple partial seizures, *CPS* complex partial seizures, *sGTCS* secondary generalized tonic–clonic seizures, *MoCA* montreal cognitive assessment, *CI* cognitive impairment, SPS* stiff person syndrome, *CA* cerebellar ataxia, *NBC* neurobehavioral changes, *AEDs* antiepileptic drugs, *LEV* levetiracetam, *CBZ* carbamazepine, *CZP* clonazepam, *OXC* oxcarbazepine, *MRI* magnetic resonance imaging, *L* left, *R* right, *B* bilateral, *(M)TL* (medial) temporal lobe, *Amy* amygdala, *Hipp* hippocampus, *FL* frontal lobe, ^*18*^*F-FDG-PET/CT* 18-fluoro-deoxyglucose-positron emission tomography/computed tomography, *Tha* thalamus, *LADA* late-onset type one diabetic, *MG* myasthenia gravis, abs antibodies, *ANA* antinuclear antibodies, *TPA* thyroid peroxidase antibody, *TGA* thyroglobulin antibody, *CSF* cerebrospinal fluid, *IVMP* intravenous methylprednisolone, *IVIG* intravenous immunoglobulin, *MF* mycophenolate mofetil, *NA* not available

#### Patient 1: typical LE

**Fig. 1:**
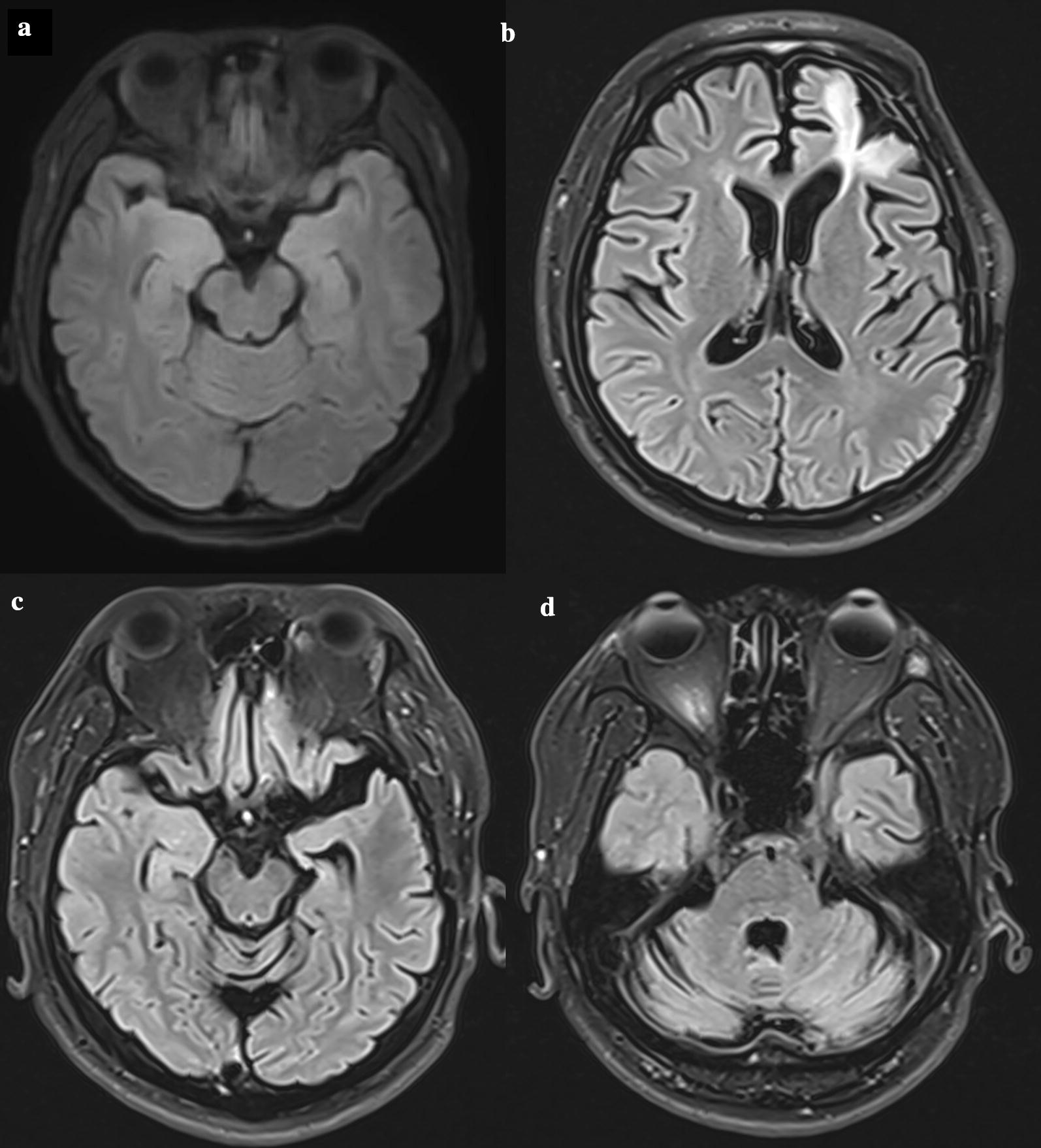
Axial magnetic resonance imaging (MRI) findings of patient 1 and patient 2 in fluid-attenuated inversion recovery (FLAIR) sequence. **a** Patient 1, a 35-year-old Chinese female, was diagnosed with typical limbic encephalitis, and MRI showed increased FLAIR signals of the right medial temporal lobe, including the amygdala and hippocampus. **b**–**d** Patient 2, a 35-year-old male, was characterized by stiff-person syndrome, cerebellar ataxia and intractable epilepsy. MRI indicated postoperative changes in the left frontal lobe, volume reduction in the left temporal lobe and hippocampus, and encephalatrophy, especially in the bilateral cerebellum

**Fig. 2:**
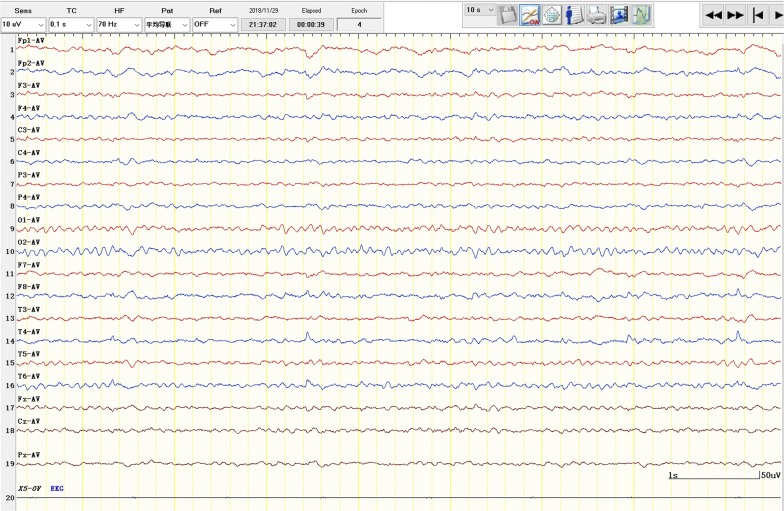
Interictal phase electroencephalogram of patient 1. There were frequent sharp waves and slow waves in the right temporal regions in the interictal phase

**Fig. 3:**
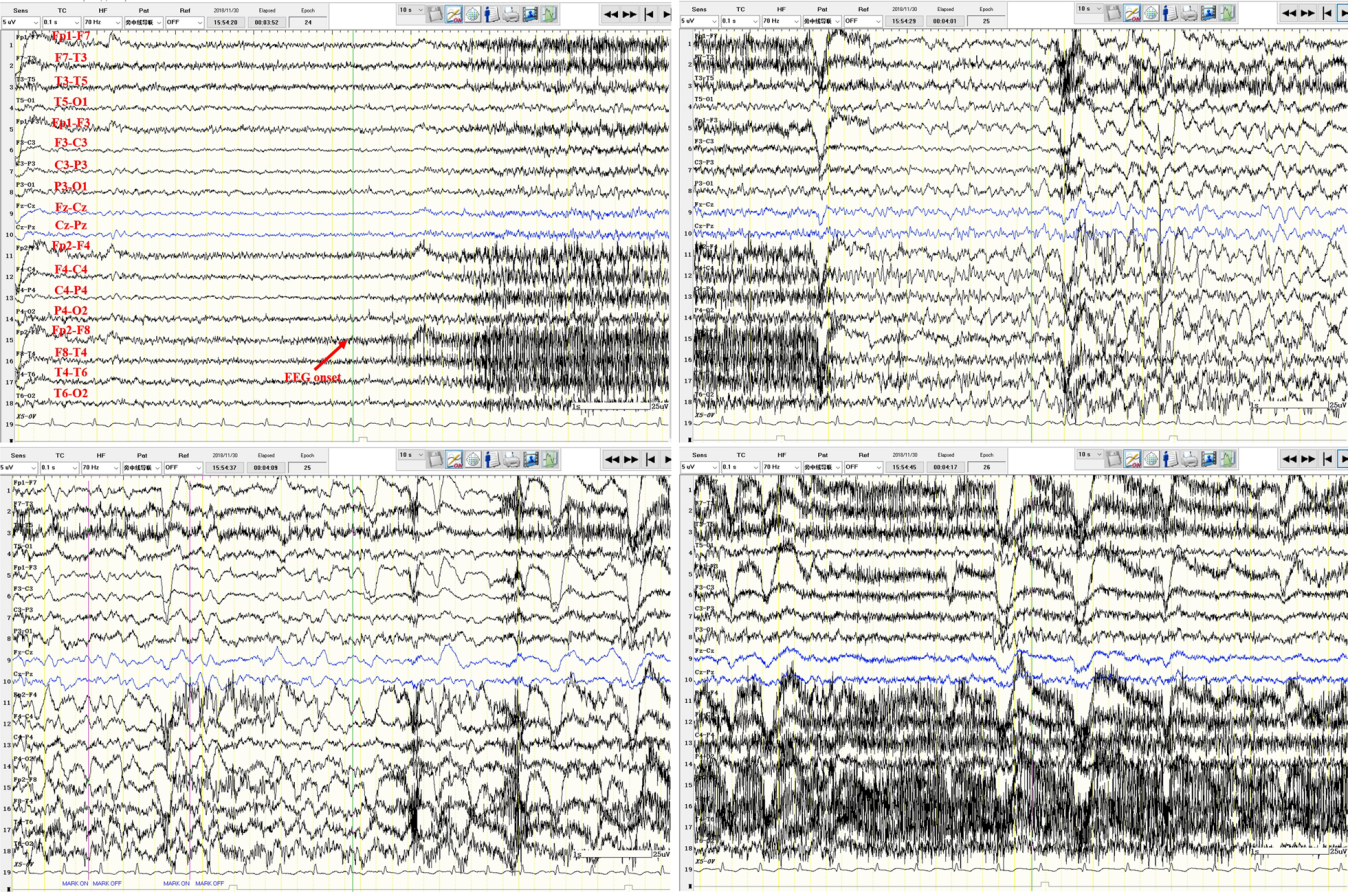
Ictal phase electroencephalogram of patient 1. One clinical seizure was detected, which presented as a nauseous feeling and oropharyngeal automatism with impaired awareness, and the seizure lasted approximately 50 s second before sudden movement interruption of the patient, EEG showed widespread low voltage in the right leads, followed by rapid rhythmic changes with low amplitude in the right middle-posterior temporal region initially, and spreading to the right middle temporal region 2 s later. The amplitude increased and the frequency decreased gradually and then spread to adjacent leads, accompanied by electromyogram interference and motion artifacts

A 35-year-old female reported sudden onset of nocturnal generalized tonic–clonic seizures (GTCSs) 3 months ago; these GTCSs lasted approximately 1 min, with 5 episodes in total. In addition, she also had an inexplicable sense of fear, several times per day. She received adequate doses of levetiracetam, and the effect was not obvious. During the past 2 months, she experienced 3–4 conscious nauseous sensations per week. Initial neurological examination revealed only a decrease in calculation capacity. The Montreal Cognitive Assessment (MoCA) score was 22 (primary school degree) on a scale ranging from 0 to 30. Routine examinations of blood and CSF were normal. Comprehensive onconeural and neuronal surface Abs screening in the serum and CSF, detected by the cell-based transfection immunofluorescence assay method, showed only positive anti-GAD65 Abs. Brain magnetic resonance imaging (MRI) showed abnormal signals of the right medial temporal lobe (Fig. 1a). The long-term video electroencephalogram (EEG) showed significant frequent sharp waves, slow waves and sharp-slow complex waves in the right frontal and temporal regions during the interictal phase (Fig. 2). During the ictal phase, we found that the EEG rhythmic changes first appeared in the right central, parietal, posterior temporal and midline regions, accompanied by clinical seizures approximately 1 s later, manifested as a nauseous feeling and oropharyngeal automatism with impaired awareness, and the seizures lasted approximately 50 s (Fig. 3). The frequency of the above episodes was 2–3 times/day. Therefore, the patient was diagnosed with anti-GAD65 LE, and a 5-day course of IVMP, 500 mg/day, was initiated and gradually reduced. Subsequently, she took prednisone (1 mg/kg/day) and levetiracetam (750 mg/q12h) for maintenance therapy. The clinical improvement was remarkable. Four months later, she received a 5-day course of IVIG, 400 mg/kg/day, for consolidation therapy. Currently, she takes only levetiracetam and feels nauseous occasionally.

#### Patient 2: SPS, CA and intractable epilepsy

The patient is a 35-year-old male. His mother had suffered hyperthyroidism. He was also diagnosed with hyperthyroidism at 15 years old and developed nocturnal GTCSs 2 years later. These attacks lasted 4–5 min with a frequency of twice per month. At 24 years of age, he gradually developed barylalia and gait instability, and these symptoms progressively deteriorated. The second form of seizure occurred at 25 years of age and manifested as right-side deflection of the mouth, eyeball and head in an unconscious state, accompanied by oropharyngeal automatism and groping action. The seizure lasted 2–3 min and also occurred at night, with 1–2 seizures/year. Three years later, the frequency of the second type increased to once per week. He was diagnosed with late-onset type one diabetes at the age of 31, and blood sugar was well controlled. Three years ago, he underwent an excision of the epileptogenic focus in the left frontal lobe due to intractable epilepsy. After the operation, he received adequate doses of levetiracetam, clonazepam and carbamazepine, and although the first type of seizure disappeared, no improvement was achieved in the second type. Two months ago, the patient appeared to have a third form of seizure, which was characterized as a sudden trance and interruption of actions, lasting 2–20 min, with a frequency of one per day. In recent days, he suffered paroxysmal muscle stiffness and limb rigidity, accompanied by soreness of the lumbar muscles and difficulty in lying flat; these symptoms were prone to occur when stimulated by environmental factors. In addition, he occasionally developed palpitations, sweats and breathing difficulties and became irritable and depressed.

On neurological examination, he exhibited bilateral exophthalmos, dysarthria and left blepharospasm. The muscular tension and tendon reflex of the four limbs were decreased. He could not stand still and complete the finger-nose tests. The MoCA score was 22 (undergraduate degree). Routine blood examinations suggested that he had multiple kinds of autoimmune Abs simultaneously (seen in Table 1). Routine CSF analysis was normal. The onconeural and neuronal surface Abs test showed positive anti-GAD65 Abs in both serum and CSF. The brain MRI indicated postoperative changes and encephalatrophy (Fig. 1b–d). Electromyography showed continuous release of motor unit action potentials of the paraspinal muscles and rectus abdominis in a quiet state, which were obviously inhibited after intravenous diazepam, supporting the diagnosis of SPS.

A 5-day course of IVIG, 400 mg/kg/day, was initiated; subsequently, he took mycophenolate mofetil (gradually increased to 1500 mg/q12h), levetiracetam (250 mg/q12h), clonazepam (1 mg/q12h) and carbamazepine (200 mg/q12h) for long-term maintenance therapy. The above symptoms improved significantly. One month later, he received another 5-day course of IVIG for consolidation therapy. Three months later, the SPS had almost disappeared, the ataxia improved, he could walk by himself, and the number of seizures was reduced by more than 50%.

### Patients from previous literatures

Seventy references were identified, and the included patients were all diagnosed with anti-GAD65 AE. In Table 2, we present the gathered data of all patients with different clinical manifestations and found that the effectiveness of IVMP was 45.56% (41/90) and that of IVIG was 36.71% (29/79). There was no statistically significant difference between IVMP and IVIG. In addition, 35 patients received IVMP and IVIG combination therapy, but only 42.86% (15/35) experienced an improvement in their clinical symptoms.

**Table 2:** IVMP and IVIG effectiveness of total patients in the previous 70 references

	Effective number	Ineffective number	*p*
IVMP	41	49	
IVIG	29	50	0.244

Differences were evaluated by Chi square test

*IVMP* intravenous methylprednisolone, *IVIG* intravenous immunoglobulin

We further divided patients according to their typical clinical manifestations. In Table 3, we present data on the patients who manifested seizures, 42.65% (29/68) of whom were responsive to IVMP and 32.69% (17/52) responded to IVIG (in comparison with Table 2, in Table 3, we added an extra patient, whose seizures were not improved with IVMP, while IVMP was beneficial for CA) [28]. Among patients with SPS, 60.00% (3/5) benefited from IVMP, and 77.78% (7/9) gained amelioration from IVIG. In patients with CA, the effectiveness of IVMP and IVIG was 28.57% (2/7) and 55.56% (5/9), respectively (in Table 3, an extra patient was added in comparison with Table 2 [28]). In these above patients from different subgroups, there was no difference in therapeutic effect between the two kinds of treatment. Furthermore, in these AE patients who manifested seizures or CA, the effectiveness of combination therapy was 44.83% (13/29) and 25.00% (1/4), respectively. In our analysis, only one SPS patient received combination therapy but acquired no benefits [7].

**Table 3:** IVMP and IVIG effectiveness of patients with different clinical manifestations in the previous 70 references

	Patients with seizures			Patients with SPS			Patients with CA			Patients with tumors		
	EN	IEN	*p*	EN	IEN	*p*	EN	IEN	*p*	EN	IEN	*p*
IVMP	29	39		3	2		2	5		5	12	
IVIG	17	35	0.266	7	2	0.580^*^	5	4	0.358^*^	8	11	0.429

Differences were evaluated by Chi square test or Fisher’s exact test*

*EN* effective numbers, *IEN* ineffective numbers, *IVMP* intravenous methylprednisolone, *IVIG* intravenous immunoglobulin

As shown in Table 3 and Additional file 2: Table S1, of the 29 anti-GAD65 AE patients with tumors, 16 (55.17%) were male, and the median age was 61.8 years (IQR: 55.0–73.0 years). It should be pointed out that a 5-year-old girl was diagnosed with AE 13 months after hematopoietic stem cell transplantation (because of pineoblastoma); thus, the tumor and the AE may not have been directly related [36]. The median age of the remaining 28 patients increased to 63.9 years (IQR: 57.0–73.0 years), much higher than the median age of 48 years for the 106 anti-GAD65 patients without tumors in a previous study [7]. The most common symptom was SPS (11/29, 37.93%), followed by LE and nonlimbic AE (8/29 and 4/29, respectively). Among these patients, the therapeutic effect was dismal, with 29.41% and 42.11% improvement rates in symptoms for IVMP and IVIG, respectively, and no significant difference was found. Moreover, the combination therapy was effective for only 20.00% (2/10) of patients. In addition, 21 patients (72.41%) had oncological treatment (surgery, chemotherapy, and radiotherapy alone or combined). Other clinical data are listed in Additional file 2: Table S1.

It should be mentioned that we cannot compare the efficiency of combination therapy with monotherapy because of their statistical overlaps.

## Discussion

Screening for anti-GAD65 Abs has been widely reported among type 1 diabetic patients. However, increased awareness of neurologists in considering patients with anti-GAD65 Abs remains challenging. Among adult epilepsy patients with unknown etiology, three recent studies found that 1.7% (7/416) [37], 16.1% (18/112) [38], and 21.7% (5/23) of patients were positive for serum anti-GAD65 Abs [39]. The anti-GAD65-Ab positive rate was 17.0% (9/53) in LE patients [40]. Hence, the incidence of anti-GAD65 diseases may be seriously underestimated in practice. Here, we have described the clinical information of six anti-GAD65 AE patients in our cohort, five of whom were diagnosed after years of repeated medical consultations. Therefore, it should be noted that the time from symptom onset to immunotherapy was generally very long due to the lack of awareness, and some patients, including one of ours, even underwent brain tissue resection for drug-resistant epilepsy, which usually led to unsatisfactory outcomes [11, 41, 42].

Currently, there is no consensus on the treatment of anti-GAD65 AE. However, we believe that anti-GAD65 Abs are unique compared with other onconeural Abs. Patients with neurological syndromes associated with anti-GAD65 Abs are not typically considered at very high risk for cancer [7], and their prognosis seems to be very different from that of other traditional onconeural Abs [6, 25]. Jones et al. proved that the detection of anti-GAD65 Abs in adult autoimmune CA patients may predict relatively better immunotherapy response and neurological outcomes, similar to those of patients seropositive for neuronal surface Abs [6]. In contrast, the beneficial effects of immunotherapy on seizure frequency and cognition can be acquired only in paraneoplastic Ab-positive LE patients but not obviously in anti-GAD65 LE patients [25]. Furthermore, according to considerable research efforts devoted to the comparison of AE patients with anti-GAD65 Abs and those with anti-voltage gated potassium channel Abs, researchers have verified that the former tend to be seriously resistant to AEDs and immunotherapy, resulting in a chronic situation [12, 40, 43].

Gagnon et al. compiled an excellent review of 31 articles, describing the detailed treatment regimens of patients [8]. In contrast, our study considered only the response of patients to IVMP or IVIG due to the complexity of clinical treatments, and we attempted to analyze the effectiveness of the chosen treatments for patients from 70 previous articles. In patients who received IVMP, the effective rate was 45.56% (41/90), which was slightly higher than that of 36.71% (29/79) for patients who received IVIG. In the study of Gagnon et al. [8], the favorable outcome rates were 46.15% (6/13) for IVMP and 50.00% (2/4) for IVIG, and the effective rate for IVMP was similar to ours; however, the number of patients in our study was much more than that in their study because we searched more comprehensive references and included more patients. In patients with seizures, the response rates of IVMP and IVIG were 42.65% (29/68) and 32.69% (17/52), respectively, which were similar when counting all patients. Although we cannot recommend which method is more effective because no significant difference between IVMP and IVIG was found, importantly, it is very difficult for these patients to become seizure free from only AEDs [12, 13], and immunotherapy is currently still indispensable. In patients with SPS or CA, IVIG seemed to have a slightly better therapeutic effect than IVMP (77.78% vs. 60.00%; 55.56% vs. 28.57%). Consistent with our results, in a randomized, double-blinded, placebo-controlled crossover trial in anti-GAD65-Ab-positive SPS patients, IVIG resulted in significant improvements in objective stiffness parameters and activities of daily living [44], and corticosteroids or other therapies may be very disappointing [45]. However, as for CA, a study of 118 CA patients (including 41 anti-GAD65-Ab-positive cases) drew a different conclusion from ours, suggesting that corticosteroids may be the best regimen [6]. Considering the small sample size and the heterogeneity of subjects, further research is needed. Theoretically, combined therapy should be better than monotherapy, but the effective rate was disappointing in our research, with only 15/35 (42.86%) in all patients and 13/29 (44.83%), 0/1 (0%), and 1/4 (25.00%) in epilepsy, SPS and CA patients, respectively. A similar conclusion was obtained in the study of Gagnon et al. where steroids plus IVIG or plasma exchange had beneficial effects on only 6/18 (33.33%) anti-GAD65 LE patients [8]. One important reason contributing to the results is that patients who received combined immunotherapy were usually in serious conditions. Although we cannot make comparisons with monotherapy due to statistical overlaps, the method is still highly recommendable and very promising.

In our study, we also observed 29 anti-GAD65 AE patients who had coexisting tumors; in general, their reactions to immunotherapy were unsatisfactory. Ten of them received combined treatment of IVMP and IVIG, but only two benefited. Among the remaining 19 patients, the effectiveness of IVMP and IVIG was 55.56% (5/9) and 72.73% (8/11), respectively (one patient received IVMP and IVIG successively, not in combination); however, when calculated together, the improvement rates of IVMP and IVIG slumped to 29.41% (5/17) and 42.11% (8/19), respectively. Interestingly, a good outcome may be predicted for patients who had coexisting thymoma; only one out of 8 did not respond to immunotherapy, but the symptoms could be relieved by benzodiazepines or baclofen [46]. In contrast, only two out of 11 lung cancer patients acquired temporary improvement with immunotherapy, and one died in a short period of time [47]. The results are consistent with a previous observation, which found that 46.67% (7/15) of anti-GAD65-associated paraneoplastic neurological syndrome patients acquired clinical improvement or stability under various immunotherapy treatments, and amelioration occurred in only 4 thymic tumor patients, while only 1/6 of lung cancer patients was stable, and the rest were all deteriorated [7]. In addition to the older age compared with tumor-negative anti-GAD65 AE patients, which probably led to poor prognosis, tumor treatment is also a factor that needs to be considered. Among patients undergoing active treatment of primary tumors, 13/21 (61.90%) gained benefits from immunotherapy, while no patients improved if the tumor was not managed. Considering the adverse influences of particular tumor types, old age and passive tumor treatment on prognosis, the underlying tumors should be screened in some conditions early [7], and elaborate studies involving various factors are critically needed to guide future therapy.

There was no denying that some anti-GAD65 patients completely recovered without immunotherapy; however, they were reported just in scattered case reports [12, 19, 23, 48, 49]. We firmly believe that this AE was characterized as a form of chronic, non-remitting disorder in most cases, which was consistent with the standpoint from other researchers [8, 11, 40]. Several medical centers also tried to summarize their own treatment experiences and expected to offer the best treatment plan, but all ended in failure [9, 11, 28, 40, 48]. Seven anti-GAD65 LE patients received monthly high-intensity immunotherapy, with a median total methylprednisolone equivalent dose up to 18 g, but none became seizure free [40]. Malter et al. retrospectively analyzed the complex treatment regimens of 13 anti-GAD65 epilepsy patients and found that the seizure and cognitive response to immunotherapies were poor, with the most frequently achieved seizure response (≥ 50% reduction) occurring in only 45% of patients under intensive corticosteroid treatment (median 19 g methylprednisolone equivalent) [11]. Pittock et al. found that the benefits from a trial of immunotherapies in 27 patients with anti-GAD65 Abs were temporary [9]. Identical conclusions were obtained in 12 other patients [28, 48], making the routine use of immunotherapy debatable. Nevertheless, anti-GAD65-associated SPS is likely to be an exception because more than 70% of patients can obtain relief from IVIG, and notably, the abovementioned clinical trial further clarifies the validity of IVIG [44].

Our study also had some limitations. First, the retrospective analysis of the literature had the following inherent limitations: the diverse vocabularies could have led to a certain degree of misinterpretation; the description of clinical features, investigations, and outcomes also differed from one article to another. Second, we abandoned some related literature because we could not obtain the prognosis after immunotherapy, leading to statistical bias. Third, different hospitals had their own experience in choosing IVMP, IVIG or others, and the absence of IVMP or IVIG therapy did not mean that the patient was insensitive to it. Fourth, we excluded many patients who received combination therapy (IVMP/IVIG and others) because we could not judge which drug had an effect. Fifth, there was a certain ineluctable subjectivity in our judgment of reactivity to therapy. Sixth, there were significant differences in the follow-up time of patients, and we focused only on whether the clinical symptoms were improved after immunotherapy, leading to the inability to judge the effective time of IVIG and IVMP treatment. Although many patients were initially sensitive to immunotherapy, they will relapse inevitably after a period of time [13, 24, 36, 41, 48, 50–54], which we considered may be a short-term outcome. Considering the shortcomings of our research and limitations in this field, therapeutic strategies cannot be reasonably recommended currently except IVIG for anti-GAD65-associated SPS, and long-term immunotherapy may be the best option.

## Conclusions

Here, we reported six anti-GAD65 AE patients and found that they all achieved clinical improvements in a short period of time after immunotherapy. However, by summarizing the therapeutic effects of previous patients, we have confirmed that this AE usually has a poor response to IVMP or IVIG, except anti-GAD65-associated SPS. Larger prospective studies enrolling large numbers of patients are required to identify the optimal therapeutic strategy.

## Supplementary information

**Additional file 1.** References included in the statistics. We conducted a search on PubMed for articles up to April 2019 and finally included 70 references. The article type, clinical characteristics of each patient, and whether the patient received combined therapy are noted.

**Additional file 2: Tabe S1.** Clinical data of 29 patients who had coexisting tumors in previous references. The clinical information, concomitant tumor, tumor therapy and response to immunotherapy of each patient are listed.

## Data Availability

The datasets supporting the conclusions of this article are available when contacting authors.

## Abbreviations

GAD65: Glutamic acid decarboxylase 65
Abs: Antibodies
CSF: Cerebrospinal fluid
SPS: Stiff-person syndrome
CA: Cerebellar ataxia
LE: Limbic encephalitis
AE: Autoimmune encephalitis
IVMP: Intravenous methylprednisolone
IVIG: Intravenous immunoglobulin
AEDs: Antiepileptic drugs
GTCSs: Generalized tonic–clonic seizures
MoCA: Montreal Cognitive Assessment
MRI: Magnetic resonance imaging
EEG: Electroencephalogram
