# Supplementary material for: Intravenous methylprednisolone or immunoglobulin for anti-glutamic acid decarboxylase 65 antibody autoimmune encephalitis: which is better?
Source: BMC Neurosci. 2020 Mar 30;21:13. doi: 10.1186/s12868-020-00561-9 (PMC7106675; doi:10.1186/s12868-020-00561-9)
Supplement: Supplementary file 1 — Additional file 1. References included in the statistics. We conducted a search on PubMed for articles up to April 2019 and finally included 70 references. The article type, clinical characteristics of each patient, and whether the patient received combined therapy are noted. [file 12868_2020_561_MOESM1_ESM.docx]

**Supplementary Material**

We conducted a search on PubMed for articles up to April 2019 and using the title/abstract “encephalitis” and “GAD” or “glutamic acid decarboxylase”. A total of 133 references were retrieved. 49 articles, including 38 case (series) reports and 11 research articles, were screened out.

The black color represents case (series) reports and the red color represents research articles. At the end of these references, if we marked “Epilepsy, SPS or CA”, it means that patients in theses references presented with seizures, SPS or CA; if we marked “Tumor”, it means that patients in theses references coexisted with tumors; if we marked “Combination therapy”, it means that patients in theses references received IVMP and IVIG combination therapy.

1. Hansen N, Ernst L, Rüber T, et al. Pre- and long-term postoperative courses of hippocampus-associated memory impairment in epilepsy patients with antibody-associated limbic encephalitis and selective amygdalohippocampectomy. Epilepsy Behav. 2018. 79: 93-99. Epilepsy

2. Mäkelä KM, Hietaharju A, Brander A, Peltola J. Clinical Management of Epilepsy With Glutamic Acid Decarboxylase Antibody Positivity: The Interplay Between Immunotherapy and Anti-epileptic Drugs. Front Neurol. 2018. 9: 579. Epilepsy; Combination therapy

3. Incecik F, Herguner OM, Besen S, Yılmaz M. Autoimmune encephalitis associated with glutamic acid decarboxylase antibodies: a case series. Acta Neurol Belg. 2018. 118(3): 411-414. Epilepsy; Combination therapy

4. Nakajima H, Nakamura Y, Inaba Y, et al. Neurologic disorders associated with anti-glutamic acid decarboxylase antibodies: A comparison of anti-GAD antibody titers and time-dependent changes between neurologic disease and type I diabetes mellitus. J Neuroimmunol. 2018. 317: 84-89. Tumor; Epilepsy; SPS; CA; Combination therapy

5. Triplett J, Vijayan S, MacDonald A, et al. Fulminant Anti-GAD antibody encephalitis presenting with status epilepticus requiring aggressive immunosuppression. J Neuroimmunol. 2018. 323: 119-124. Epilepsy; Combination therapy

6. Feyissa AM, López CAS, Britton JW. Antiepileptic drug therapy in patients with autoimmune epilepsy. Neurol Neuroimmunol Neuroinflamm. 2017. 4(4): e353. Epilepsy

7. M'zahem A, Meziani A, Taghane N, Boulefkhad A, Khellaf S, Hamri A. Limbic encephalitis associated with glutamic acid decarboxylase antibodies in a young adolescent. Rev Neurol (Paris). 2016. 172(4-5): 327-8. Epilepsy

8. Gardner R, Rangaswamy R, Peng YY. Correlations of Clusters of Non-Convulsive Seizure and Magnetic Resonance Imaging in a Case With GAD65-Positive Autoimmune Limbic Encephalitis. J Clin Med Res. 2016. 8(8): 616-22. Epilepsy; Combination therapy

9. Kopczak A, Schumacher AM, Nischwitz S, Kümpfel T, Stalla GK, Auer MK. GAD antibody-associated limbic encephalitis in a young woman with APECED. Endocrinol Diabetes Metab Case Rep. 2017. 2017. Epilepsy

10. Elisak M, Krysl D, Hanzalova J, et al. The prevalence of neural antibodies in temporal lobe epilepsy and the clinical characteristics of seropositive patients. Seizure. 2018. 63: 1-6. Epilepsy

11. Nagai K, Maekawa T, Terashima H, Kubota M, Ishiguro A. Severe anti-GAD antibody-associated encephalitis after stem cell transplantation. Brain Dev. 2019. 41(3): 301-304. Tumor; Epilepsy; Combination therapy

12. Ariño H, Höftberger R, Gresa-Arribas N, et al. Paraneoplastic Neurological Syndromes and Glutamic Acid Decarboxylase Antibodies. JAMA Neurol. 2015. 72(8): 874-81. Tumor; Epilepsy; SPS; CA; Combination therapy

13. Popkirov S, Sebastian S, Ismail FS, Wellmer J. Latent autoimmune diabetes and limbic encephalitis with antibodies against glutamic acid decarboxylase. J Diabetes. 2018. 10(4): 338-340. Epilepsy

14. Niehusmann P, Widman G, Eis-Hübinger AM, et al. Non-paraneoplastic limbic encephalitis and central nervous HHV-6B reactivation: Causality or coincidence. Neuropathology. 2016. 36(4): 376-80. Epilepsy

15. Lu J, Samson S, Kass J, Ram N. Acute psychosis in a pregnant patient with Graves' hyperthyroidism and anti-NMDA receptor encephalitis. BMJ Case Rep. 2015. 2015.

16. Witt JA, Vogt VL, Widman G, Langen KJ, Elger CE, Helmstaedter C. Loss of Autonoetic Awareness of Recent Autobiographical Episodes and Accelerated Long-Term Forgetting in a Patient with Previously Unrecognized Glutamic Acid Decarboxylase Antibody Related Limbic Encephalitis. Front Neurol. 2015. 6: 130. Epilepsy

17. Incecik F, Hergüner OM, Yıldızdaş D, Horoz O, Besen S. Limbic encephalitis with antibodies to glutamic acid decarboxylase presenting with brainstem symptoms. Ann Indian Acad Neurol. 2015. 18(2): 243-5.

18. Grilo E, Pinto J, Caetano JS, et al. Type 1 diabetes and GAD65 limbic encephalitis: a case report of a 10-year-old girl. J Pediatr Endocrinol Metab. 2016. 29(8): 985-90. Epilepsy

19. Akaishi T, Jin K, Kato K, et al. [Clinical characteristics of four patients with temporal lobe epilepsy associated with elevated anti-GAD antibodies]. Rinsho Shinkeigaku. 2015. 55(11): 804-9. Epilepsy; Combination therapy

20. Vale TC, Pedroso JL, Alquéres RA, Dutra LA, Barsottini OG. Spontaneous downbeat nystagmus as a clue for the diagnosis of ataxia associated with anti-GAD antibodies. J Neurol Sci. 2015. 359(1-2): 21-3. CA

21. García GME, Castrillo SM, Morales IG, Di CSD, Dolado AM. Acute amnesia and seizures in a young female. Epileptic Disord. 2013. 15(4): 455-60. Epilepsy

22. Bonello M, Larner AJ, Marson AG. Profound amnesia after temporal lobectomy: an autoimmune process resembling patient h.m. Case Rep Neurol. 2014. 6(3): 251-5. Epilepsy

23. Bigi S, Hladio M, Twilt M, Dalmau J, Benseler SM. The growing spectrum of antibody-associated inflammatory brain diseases in children. Neurol Neuroimmunol Neuroinflamm. 2015. 2(3): e92. Epilepsy; Combination therapy

24. Kojima G, Inaba M, Bruno MK. PET-positive extralimbic presentation of anti-glutamic acid decarboxylase antibody-associated encephalitis. Epileptic Disord. 2014. 16(3): 358-61. Tumor; Epilepsy

25. Monnerat BZ, Velasco TR, Nakano FN, Júnior AV, Martins AP, Sakamoto AC. Opercular myoclonic-anarthric status epilepticus due to glutamic acid decarboxylase antibody-associated encephalitis. Epileptic Disord. 2013. 15(3): 342-6. Epilepsy

26. Mishra N, Rodan LH, Nita DA, Gresa-Arribas N, Kobayashi J, Benseler SM. Anti-glutamic Acid decarboxylase antibody associated limbic encephalitis in a child: expanding the spectrum of pediatric inflammatory brain diseases. J Child Neurol. 2014. 29(5): 677-83. Epilepsy

27. Millet C, van Pesch V, Sindic CJ. Idiopathic limbic encephalitis associated with antibodies to glutamic acid decarboxylase. Acta Neurol Belg. 2015. 115(2): 165-7. Epilepsy

28. Markakis I, Alexopoulos H, Poulopoulou C, et al. Immunotherapy-responsive limbic encephalitis with antibodies to glutamic acid decarboxylase. J Neurol Sci. 2014. 343(1-2): 192-4. Epilepsy

29. Georgieva Z, Parton M. Cerebellar ataxia and epilepsy with anti-GAD antibodies: treatment with IVIG and plasmapheresis. BMJ Case Rep. 2014. 2014. Epilepsy; CA

30. Farooqi MS, Lai Y, Lancaster E, Schmitt SE, Sachais BS. Therapeutic plasma exchange and immunosuppressive therapy in a patient with anti-GAD antibody-related epilepsy: quantification of the antibody response. J Clin Apher. 2015. 30(1): 8-14. Epilepsy; Combination therapy

31. Matà S, Muscas GC, Naldi I, et al. Non-paraneoplastic limbic encephalitis associated with anti-glutamic acid decarboxylase antibodies. J Neuroimmunol. 2008. 199(1-2): 155-9. Epilepsy

32. Carra-Dalliere C, Thouvenot E, Bonafé A, Ducray F, Touchon J, Charif M. [Anti-GAD antibodies in paraneoplastic cerebellar ataxia associated with limbic encephalitis and autonomic dysfunction]. Rev Neurol (Paris). 2012. 168(4): 363-6. Tumor; CA

33. Blanc F, Ruppert E, Kleitz C, et al. Acute limbic encephalitis and glutamic acid decarboxylase antibodies: a reality. J Neurol Sci. 2009. 287(1-2): 69-71. Epilepsy

34. Cianci V, Labate A, Lanza P, et al. Non-paraneoplastic limbic encephalitis characterized by mesio-temporal seizures and extratemporal lesions: a case report. Seizure. 2010. 19(7): 446-9. Epilepsy

35. Boronat A, Sabater L, Saiz A, Dalmau J, Graus F. GABA(B) receptor antibodies in limbic encephalitis and anti-GAD-associated neurologic disorders. Neurology. 2011. 76(9): 795-800. Tumor; Epilepsy; CA; Combination therapy

36. Cikrikçili U, Ulusoy C, Turan S, et al. Non-convulsive status epilepticus associated with glutamic acid decarboxylase antibody. Clin EEG Neurosci. 2013. 44(3): 232-6. Epilepsy

37. Dayalu P, Teener JW. Stiff Person syndrome and other anti-GAD-associated neurologic disorders. Semin Neurol. 2012. 32(5): 544-9. CA

38. Marchiori GC, Vaglia A, Vianello M, Bardin PG, Giometto B. Encephalitis associated with glutamic acid decarboxylase autoantibodies. Neurology. 2001. 56(6): 814. Epilepsy

39. Malter MP, Helmstaedter C, Urbach H, Vincent A, Bien CG. Antibodies to glutamic acid decarboxylase define a form of limbic encephalitis. Ann Neurol. 2010. 67(4): 470-8. Epilepsy; SPS

40. Lopez-Sublet M, Bihan H, Reach G, et al. Limbic encephalitis and type 1 diabetes with glutamic acid decarboxylase 65 (GAD65) autoimmunity: improvement with high-dose intravenous immunoglobulin therapy. Diabetes Metab. 2012. 38(3): 273-5. Epilepsy

41. Lin JJ, Lin KL, Hsia SH, Wang HS, Chou IJ, Lin YT. Antiglutamic acid decarboxylase antibodies in children with encephalitis and status epilepticus. Pediatr Neurol. 2012. 47(4): 252-8. Epilepsy; Combination therapy

42. Kanter IC, Huttner HB, Staykov D, et al. Cyclophosphamide for anti-GAD antibody-positive refractory status epilepticus. Epilepsia. 2008. 49(5): 914-20. Epilepsy

43. Giometto B, Nicolao P, Macucci M, Tavolato B, Foxon R, Bottazzo GF. Temporal-lobe epilepsy associated with glutamic-acid-decarboxylase autoantibodies. Lancet. 1998. 352(9126): 457. Epilepsy

44. Korff CM, Parvex P, Cimasoni L, et al. Encephalitis associated with glutamic acid decarboxylase autoantibodies in a child: a treatable condition. Arch Neurol. 2011. 68(8): 1065-8. Epilepsy

45. Saiz A, Blanco Y, Sabater L, et al. Spectrum of neurological syndromes associated with glutamic acid decarboxylase antibodies: diagnostic clues for this association. Brain. 2008. 131(Pt 10): 2553-63. Tumor; Epilepsy; Combination therapy

46. Saidha S, Murphy S, Ronayne A, McCarthy P, Hennessy MJ, Counihan T. Treatment of anti-glutamic acid decarboxylase antibody-associated limbic encephalitis with mycophenolate mofetil. J Neurol. 2010. 257(6): 1035-8. Epilepsy

47. Najjar S, Pearlman D, Najjar A, Ghiasian V, Zagzag D, Devinsky O. Extralimbic autoimmune encephalitis associated with glutamic acid decarboxylase antibodies: an underdiagnosed entity. Epilepsy Behav. 2011. 21(3): 306-13. Epilepsy; Combination therapy

48. Mirabelli-Badenier M, Morana G, Pinto F, et al. Anti-glutamic acid decarboxylase limbic encephalitis without epilepsy evolving into dementia with cerebellar ataxia. Arch Neurol. 2012. 69(8): 1064-6. CA; Combination therapy

49. Mazzi G, Roia DD, Cruciatti B, Matà S, Catapano R. Plasma exchange for anti-GAD associated non paraneoplastic limbic encephalitis. Transfus Apher Sci. 2008. 39(3): 229-33. Epilepsy

A back-search of reference lists from retrieved publications was also conducted to identify other potentially relevant articles, then, other 21 articles, including 13 case (series) reports and 8 research articles, were also included.

The black color represents case (series) reports and the red color represents research articles. At the end of these references, if we marked “Epilepsy, SPS or CA”, it means that patients in theses references presented with seizures, SPS or CA; if we marked “Tumor”, it means that patients in theses references coexisted with tumors; if we marked “Combination therapy”, it means that patients in theses references received IVMP and IVIG combination therapy.

1. Pittock SJ, Yoshikawa H, Ahlskog JE, et al. Glutamic acid decarboxylase autoimmunity with brainstem, extrapyramidal, and spinal cord dysfunction. Mayo Clin Proc. 2006. 81(9): 1207-14.

2. Lilleker JB, Biswas V, Mohanraj R. Glutamic acid decarboxylase (GAD) antibodies in epilepsy: diagnostic yield and therapeutic implications. Seizure. 2014. 23(8): 598-602. Epilepsy; CA

3. Akman CI, Patterson MC, Rubinstein A, Herzog R. Limbic encephalitis associated with anti-GAD antibody and common variable immune deficiency. Dev Med Child Neurol. 2009. 51(7): 563-7. Epilepsy

4. Malter MP, Frisch C, Zeitler H, et al. Treatment of immune-mediated temporal lobe epilepsy with GAD antibodies. Seizure. 2015. 30: 57-63. Epilepsy

5. Falip M, Rodriguez-Bel L, Castañer S, et al. Musicogenic reflex seizures in epilepsy with glutamic acid decarbocylase antibodies. Acta Neurol Scand. 2018. 137(2): 272-276. Epilepsy

6. Iwata T, Inoue K, Mizuguchi S, Morita R, Tsukioka T, Suehiro S. Thymectomy for paraneoplastic stiff-person syndrome associated with invasive thymoma. J Thorac Cardiovasc Surg. 2006. 132(1): 196-7. Tumor; SPS

7. Tanaka H, Matsumura A, Okumura M, Kitaguchi M, Yamamoto S, Iuchi K. Stiff man syndrome with thymoma. Ann Thorac Surg. 2005. 80(2): 739-41. Tumor; SPS

8. Thomas S, Critchley P, Lawden M, et al. Stiff person syndrome with eye movement abnormality, myasthenia gravis, and thymoma. J Neurol Neurosurg Psychiatry. 2005. 76(1): 141-2. Tumor; SPS

9. Sinnreich M, Assal F, Hefft S, et al. Anti-GAD antibodies and breast cancer in a patient with stiff-person syndrome: a puzzling association. Eur Neurol. 2001. 46(1): 51-2. Tumor; SPS

10. Pandit AK, Ihtisham K, Garg A, Gulati S, Padma MV, Tripathi M. Autoimmune encephalitis: A potentially reversible cause of status epilepticus, epilepsy, and cognitive decline. Ann Indian Acad Neurol. 2013. 16(4): 577-84. Epilepsy

11. Petit-Pedrol M, Armangue T, Peng X, et al. Encephalitis with refractory seizures, status epilepticus, and antibodies to the GABAA receptor: a case series, characterisation of the antigen, and analysis of the effects of antibodies. Lancet Neurol. 2014. 13(3): 276-86. Epilepsy

12. Graus F, Saiz A, Lai M, et al. Neuronal surface antigen antibodies in limbic encephalitis: clinical-immunologic associations. Neurology. 2008. 71(12): 930-6. Epilepsy; Combination therapy

13. Baglietto MG, Mancardi MM, Giannattasio A, et al. Epilepsia partialis continua in type 1 diabetes: evolution into epileptic encephalopathy with continuous spike-waves during slow sleep. Neurol Sci. 2009. 30(6): 509-12. Epilepsy

14. Sharma A, Dubey D, Sawhney A, Janga K. GAD65 Positive Autoimmune Limbic Encephalitis: A Case Report and Review of Literature. J Clin Med Res. 2012. 4(6): 424-8. Epilepsy; Combination therapy

15. Quek AM, Britton JW, McKeon A, et al. Autoimmune epilepsy: clinical characteristics and response to immunotherapy. Arch Neurol. 2012. 69(5): 582-93. Epilepsy; Combination therapy

16. Laroumagne S, Elharrar X, Coiffard B, et al. "Dancing eye syndrome" secondary to opsoclonus-myoclonus syndrome in small-cell lung cancer. Case Rep Med. 2014. 2014: 545490. Tumor; CA

17. Lamotte G, Danaila TC, Jaillon-Rivière V, Hitier M, Defer GL. Paraneoplastic opsoclonus myoclonus with autoantibodies to glutamic acid decarboxylase. Rev Neurol (Paris). 2014. 170(1): 50-1. Tumor; CA

18. Spitz M, Ferraz HB, Barsottini OG, Gabbai AA. Progressive encephalomyelitis with rigidity: a paraneoplastic presentation of oat cell carcinoma of the lung. Case report. Arq Neuropsiquiatr. 2004. 62(2B): 547-9. Tumor; SPS

19. Kobayashi R, Kaji M, Horiuchi S, Miyahara N, Hino Y, Suemasu K. Recurrent thymoma with stiff-person syndrome and pure red blood cell aplasia. Ann Thorac Surg. 2014. 97(5): 1802-4. Tumor; SPS

20. McHugh JC, Murray B, Renganathan R, Connolly S, Lynch T. GAD antibody positive paraneoplastic stiff person syndrome in a patient with renal cell carcinoma. Mov Disord. 2007. 22(9): 1343-6. Tumor; SPS

21. Falip M,Carreño M,Miró J et al. Prevalence and immunological spectrum of temporal lobe epilepsy with glutamic acid decarboxylase antibodies.[J] .Eur. J. Neurol., 2012, 19: 827-33. Epilepsy
