# Supplementary material for: Intravenous methylprednisolone or immunoglobulin for anti-glutamic acid decarboxylase 65 antibody autoimmune encephalitis: which is better?
Source: BMC Neurosci. 2020 Mar 30;21:13. doi: 10.1186/s12868-020-00561-9 (PMC7106675; doi:10.1186/s12868-020-00561-9)
Supplement: Supplementary file 2 — Additional file 2: Tabe S1. Clinical data of 29 patients who had coexisting tumors in previous references. The clinical information, concomitant tumor, tumor therapy and response to immunotherapy of each patient are listed. [file 12868_2020_561_MOESM2_ESM.docx]

| **Additional Table S1: Clinical data of 29 patients who had coexisting tumors in previous references** | | | | | | | |
| --- | --- | --- | --- | --- | --- | --- | --- |
| **Patient (age/sex)** | **Clinical syndrome** | **Combined tumor** | **Tumor therapy** | **IVMP** | **IVIG** | **IVMP+IVIG** | **Reference (Corresponding to the supplementary file)** |
| 1, 35/F | SPS | Breast cancer | Lumpectomy, chemotherapy and radiotherapy |  | + |  | 4 |
| 2, 60/F | Brainstem encephalitis | Thymoma | Thymectomy |  |  | + | 4 |
| 3, 67/F | SPS | Thymoma, thyroid carcinoma | NA |  | + |  | 4 |
| 4, 5/F | Encephalitis | Pineoblastoma | Chemotherapy, radiotherapy, HSCT |  |  | + | 11 |
| 5, 80/M | LE | SCLC | Chemotherapy | - |  |  | 12 |
| 6, 79/M | CA | NSCLC | No treatment |  |  | - | 12 |
| 7, 67/M | PEM | Pancreatic neuroendocrine Carcinoma | Surgery |  | - |  | 12 |
| 8, 73/F | LE | NHL (Burkitt) | No treatment |  |  | - | 12 |
| 9, 70/M | LE | SCLC | No treatment |  |  | - | 12 |
| 10, 78/F | Brainstem encephalitis | Pancreatic neuroendocrine carcinoma | Surgery and chemotherapy |  | - |  | 12 |
| 11, 66/M | LE | SCLC | No treatment |  |  | - | 12 |
| 12, 37/M | LE | Thymoma | Surgery | + |  |  | 12 |
| 13, 72/M | OMS | NSCLC | Surgery and chemotherapy |  |  | - | 12 |
| 14, 47/M | SPS | SCLC | Chemotherapy | - |  |  | 12 |
| 15, 40/F | SPS | Breast cancer | Surgery and chemotherapy |  |  | - | 12 |
| 16, 66/F | Encephalitis | Colon cancer, pheochromocytoma, thymoma | Surgery, but remission | + |  |  | 24 |
| 17, 66/M | LE | SCLC | NA |  |  | - | 35 |
| 18, 57/F | CA | Carcinoid of thymus | NA | + |  |  | 35 |
| 19, 59/M | LE, CA | SCLC | Received treatment, but not clear |  | + |  | 32 |
| 20, 70/M | LE | SCLC | No treatment |  |  | - | 45 |
| 21, 85/F | SPS | Breast cancer | Surgery and antihormonal therapy | + |  |  | 9 (back-research) |
| 22, 45/M | SPS | Thymoma | Thymectomy |  | - |  | 8 (back-research) |
| 23, 57/F | SPS | Thymoma | Thymectomy |  | + |  | 7 (back-research) |
| 24, 79/F | SPS | Thymoma | Thymectomy | - | + |  | 6 (back-research) |
| 25, 53/M | SPS | Renal cell carcinoma | Surgery |  | + |  | 20 (back-research) |
| 26, 81/F | SPS | Thymoma | Surgery | + |  |  | 19 (back-research) |
| 27, 73/M | SPS | SCLC | Chemotherapy |  | + |  | 18 (back-research) |
| 28, 61/M | OMS | Carcinoma of the right pyriform sinus | Chemotherapy and radiotherapy |  | + |  | 17 (back-research) |
| 29, 65/M | OMS | SCLC | Chemotherapy | - |  |  | 16 (back-research) |

Abbreviations: GAD65, Glutamic acid decarboxylase 65; Abs, antibodies; AE, autoimmune encephalitis; IVMP, intravenous methylprednisolone; IVIG, intravenous immunoglobulin; F, female; M, male; SPS, stiff-person spectrum; NA, not available; HSCT, hematopoietic stem cell transplantation; LE, limbic encephalitis; CA, cerebellar ataxia; SCLC, small cell lung cancer; NSCLC, non-SCLC; PEM, paraneoplastic encephalomyelitis; NHL, non-Hodgkin lymphoma; OMS, opsoclonus-myoclonus syndrome.
